# Cobalt-modified palladium nanocatalyst on nitrogen-doped reduced graphene oxide for direct hydrazine fuel cell†

**DOI:** 10.1039/d1ra07099a

**Published:** 2021-12-08

**Authors:** Mir Ghasem Hosseini, Vahid Daneshvari-Esfahlan, Sigrid Wolf, Viktor Hacker

**Affiliations:** Electrochemistry Research Laboratory, Department of Physical Chemistry, Faculty of Chemistry, University of Tabriz Tabriz 51666-16471 Iran; Institute of Chemical Engineering and Environmental Technology, Graz University of Technology Inffeldgasse 25/C 8010 Graz Austria

## Abstract

Nitrogen-doped reduced graphene oxide-supported palladium–cobalt nanoparticles (PdCo NPs/NrGO NSs) are synthesized and used as a high-performance and low-cost anodic catalyst for direct hydrazine–hydrogen peroxide fuel cells. The SEM and TEM images of PdCo NPs/NrGO NSs show the uniform metal nanoparticle distribution on the NrGO NSs. The reduction of the oxygen functional groups and the doping of the nitrogen atoms in the GO framework are confirmed by FT-IR and XRD spectroscopic studies. The Pd catalysts modified by Co exhibit a higher catalytic activity, lower onset potential, better durability, and lower impedance values than unmodified Pd catalysts for the electro-oxidation of hydrazine. The kinetic studies show a first-order reaction with an activation energy of 12.51 kJ mol^−1^. A direct hydrazine–hydrogen peroxide fuel cell with PdCo NPs/NrGO NSs as anode and Pt/C as cathode provides an open circuit voltage of 1.76 V and a maximum power density of 148.58 mW cm^−2^ at 60 °C, indicating that the PdCo NPs/NrGO NSs are an economical, high performance and reliable anode catalyst for the direct hydrazine–hydrogen peroxide fuel cell.

## Abstract 2

The superior catalytic activity and stability of a novel anodic PdCo NPs/NrGO NSs for HzOR are confirmed by half and signal cell investigations.

## Graphical abstract

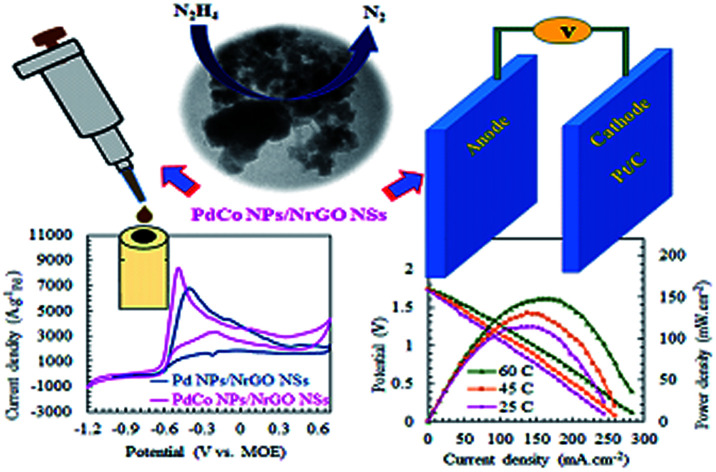

## Introduction

Cost-effective green energy technologies such as fuel cells are crucial for reducing fossil fuel consumption and mitigating climate change.^1,2^ In the field of transportable or mobile applications, direct hydrazine fuel cells (DHzFCs) are particularly suitable due to the unique properties of the hydrazine as a fuel. Hydrazine hydrate (N_2_H_4_·H_2_O) is a liquid fuel with a high energy density (9.3 W h g^−1^), easy storage and transport, a high theoretical open circuit voltage (OCV, 1.56 V), and sufficient stability under experimental ambient conditions, which made it a potential hydrogen storage medium that could be directly converted to electricity in DHzFCs.^3,4^

It is known that the use of hydrogen peroxide (H_2_O_2_) as an oxidant in DHzFCs increases the reaction kinetics at the cathode, improves the power density and the theoretical OCV.^5,6^ by using H_2_O_2_ as an oxidant in a DHzFC, the anodic, cathodic, and total cell reactions are described by eqn (1)–(3).

1 Anode: N_2_H_4_ + 4OH^−^ → N_2_ + 4H_2_O + 4e^−^*E*^0^ = −1.21 V *vs.*SHE

2 Cathode: 2H_2_O_2_ + 4H^+^ + 4e^−^ → 4H_2_O *E*^0^ = 0.92 V *vs.*SHE

3 Total: N_2_H_4_ + 2H_2_O_2_ → N_2_ + 4H_2_O *E*^0^ = 2.13 V *vs.*SHE

In a direct hydrazine–hydrogen peroxide FC (DHzHPFCs), electrons are moved from anode to cathode, whereas Na^+^ ions migrate in a contrary path *via* a Nafion membrane, and N_2_ and H_2_O produce as only products.^7–9^ However, the large-scale use of DHzHPFCs is restricted by irreversible HzOR occurred on the catalyst surface under a great overpotential. To overcome this problem, it is necessary to use the rare and precious noble metal-based catalysts.^10,11^ Up to now, a diversity of noble metals including Au,^12,13^ Pt,^14,15^ Ag,^16^ and Pd^17,18^ have utilized as electrocatalysts for hydrazine oxidation reaction (HzOR). Among these noble metals, Pd nanoparticles (NPs) are received more attention from scientific community as an anodic catalyst for DHzHPFCs due to their unique electronic properties.^19^ In order to improve the catalytic performance as well as reducing of noble metal consuming, it is crucial to architecture and modify. In this respect, different materials have designed and studied as an anodic catalysts for HzOR such as Pd nanorods,^20^ porous noble metals,^21^ bi, or multimetallic alloys/or compounds,^4,22–24^ core@shell nanostructure,^25^*etc.* According to the literature,^26–28^ Co as a non-noble metal has high catalytic activity for HzOR. In addition, Pd-alloying with Co not only decreases the use of Pd but also improves the performance of the resultant catalysts.^29,30^

The performance of PdCo nanoparticles (PdCo NPs) is further improved by incorporation on a suitable support material. A suitable support material promote the charge transfer kinetics in the catalyst due to its high electrical conductivity.^29,31,32^ Various carbon-based chemicals have been widely applied as catalyst supports.^33–38^ Among these, graphene is considered to be the most promising choice by virtue not only of its great surface area and outstanding conductivity but also because of its high durability and superior mechanical strength guaranteed by its unbeatable graphitic basal plane skeleton.^35^ The presence of oxygen-based groups at the surface of graphene can advantageously affect the attachments of metal nanoparticles on the support surface.

Moreover, theoretical investigations have shown that heteroatom doping (nitrogen, sulphur, selenium, phosphorus, *etc.*) can result in some defects on graphitic basal plane structure, which further ameliorate its physiochemical activity.^39,40^ For instance, a very high positive charge on the carbon atom adjoining to nitrogen atoms and also a positive change of Fermi energy at the apex of the Brillouin zone of rGO are reported in the literature for the nitrogen-doped reduced graphene oxide (NrGO NSs).^39–41^ NrGO NSs have been widely employed as catalyst support, or as an active material for various main electrochemical processes involving HzOR, H_2_O_2_ or O_2_ reduction, lithium-ion battery, and supercapacitor.^42,43^ Considering to the advantages of NrGO NSs as a catalyst support and good results of alloying Pd with Co transition metal, it is expected that incorporating PdCo bimetallic NPs on the NrGO NSs will be successful for improving performance in DHzHPFC applications. However, there is no report in regard with investigation of PdCo NPs/NrGO NSs in literature.

In this work, a thermal solid-state method followed by electroless reduction methods is used to synthesize of PdCo NPs decorated on the surface of NrGO NSs and designated as PdCo NPs/NrGO NSs. The structure and morphological characteristics of the PdCo NPs/NrGO NSs catalyst are analysed by Fourier-transform infrared spectroscopy (FT-IR), X-ray diffraction (XRD), scanning electron microscopy (SEM), energy-dispersive X-ray spectroscopy (EDX), and transmission microscopy (TEM). The electrocatalytic activity of PdCo NPs/NrGO NSs as an anodic electrocatalyst towards HzOR is evaluated by cyclic voltammetry (CV), chronoamperometry (CA), and electrochemical impedance spectroscopy (EIS) methods. Finally, a single FC is designed by PdCo NPs/NrGO NSs as an anode and Pt/C as a cathode to evaluate the cell performance.

## Materials and methods

### Synthesis and characterization of PdCo NPs/NrGO NSs

Graphite powder was first used for the fabrication of GO by the modified Hummer's method,^44^ Details are described in the ESI.† The resultant GO was treated by utilizing a thermal solid-state procedure to obtain NrGO NSs catalyst support. In brief, GO (0.5 g) was dispersed ultrasonically in 100 mL Millipore water for 1.0 h. Then, 0.25 M urea solution was added to the slurry stirring continuously for 2.0 h, followed by removing the solvent using a rotary evaporator. The collected solid powder was thermally treated at 800 °C for 45 min under a nitrogen atmosphere. Finally, NrGO NSs-supported bimetallic PdCo catalyst was synthesized by using an electroless reduction process using sodium borohydride as a reducing agent.^45^ By this means the precursors of Pd (PdCl_2_, 28.82 mg) and Co (CoCl_2_. 6H_2_O, 41 mg) were added into the suspension of NrGO NSs nanosheets (160 mL, 0.5 mg mL^−1^ of 2-propanol and ultrapure water (v/v: 4/1)) and the mixture was then ultrasonicated subsequently for 1 h. Following on from this the resulting slurry was heated and gradually mixed with an aqueous alkaline solution comprising of NaBH_4_ (150 mg) and NaOH (40 mg) and refluxed at 99 °C for 4.0 h. Next, the mixture was cooled under stirring for 24 h, filtered by employing Whatman paper, dried, and called PdCo NPs/NrGO NSs.

FT-IR spectrum was collected with a PerkinElmer FT-IR spectrometer to determine the existence of functional groups into GO and NrGO NSs samples. XRD patterns were achieved using an XRD-PHILIPS, PW1730 apparatus with Cu Kα radiation (*λ* = 0.154 nm). Surface morphology of synthesized nanostructures was detected by FESEM equipped to EDX microanalyzer (FESEM, MIRA3FEG-SEM, Tescan) and TEM (TEM, PHILIPS, CM 120).

### Electrochemical analysis of PdCo NPs/NrGO NSs

All the electrochemical measurements were performed using a Gamry Potentiostat/Galvanostat/ZRA (Reference 600™) in a three-electrode one-compartment electrochemical cell containing PdCo NPs/NrGO NSs on glassy carbon (GC) as a working electrode, Hg/HgO (MOE) as a reference electrode and a platinized titanium rod as a counter electrode. The working electrode was prepared by dispersing catalyst powder (5 mg) in a mixture of Millipore water, 2-propanol, and Nafion solution (5 wt%) for 2 h. Subsequently, a quantity of resultant ink was dripped on the glassy carbon electrode (GCE, 0.196 cm^2^) and dried at ambient temperature. The HzOR activity of synthesized catalysts was assessed by CV, CA, and EIS techniques. To obtain more information about the influence of temperature, sweep rate, and N_2_H_4_ concentration on the catalyst performance, the CV tests were collected in a potential range of −1.2 to 0.7 V *vs.* MOE. Additionally, the CA analyses were performed at a potential of −0.5 V *vs.* MOE. All EIS analyses were carried out in a frequency range of 10^5^ to 10^−1^ Hz with an rms amplitude of modulation potential of ±10 mV to examine the electrical resistive properties of the as-fabricated catalyst.

### Fuel cell study

The MEA was fabricated using the catalyst-coated membrane (CCM) method.^46^ Before the MEA preparation, a Nafion 117 membrane was pre-treated, as described in ESI.† For gathering cell performance data, the catalyst ink was prepared through ultrasonically dispersing of the cathodic (Pt/C (0.5 mg cm^−2^)) and anodic (PdCo NPs/NrGO NSs (1 mg cm^−2^)) catalysts in the (Millipore water + 2-propanol + Nafion solution (5 wt%)) mixture for 2 h. The resulting ink was sprayed on both sides of the pre-treated-Nafion membrane. The coated Nafion was hot-pressed at 100 °C for 1.0 min, immersed in a NaOH 2 M for 72 h, and sandwiched between two gas diffusion electrodes (GDEs). The MEA was then placed in the FC set-up to investigate its performance. Water first flowed from two electrodes for 5 h followed by a flow of 2 mol L^−1^ NaOH from an anode for 5 h to convert the H^+^ ions existence in sulfonic groups of Nafion membrane to Na^+^ ions at 40 °C. Following on from this, analysis of the DHzHPFC performance was carried out by recording the current density–potential (*I*–*V*) and current density–power density (*I*–*P*) curves using a home-made single FC. Power densities were computed by using the exerted discharging current and steady-state potential. To obtain optimum experimental conditions, these analyses were performed at different temperatures and concentrations of N_2_H_4_ and H_2_O_2_.

## Results and discussion

### Characterization of PdCo NPs/NrGO NSs

#### FT-IR study

The structural characterization of synthesized GO and NrGO NSs samples was surveyed using FT-IR spectroscopy and the results are displayed in Fig. S1.† The reduction of GO to rGO after thermally treated at 800 °C is ensured from the vanishing of oxygen functional groups in spectrum of NrGO NSs. These peaks are clearly evident in the GO spectrum (as seen in Fig. S1†). Furthermore, the embedding of nitrogen atoms into the rGO framework affirms with the appeared bands at 1187 cm^−1^. Also, the superposition of C=C and C=N vibrations leads to shifts in-plane vibration of C=C from 1624 to 1556 cm^−1^. These results are in good consistent with those reported in the literature.^47–50^ More discussion in the context of the FT-IR spectrum for catalyst support is also provided in the ESI.†

#### Morphology and structure of the PdCo NPs/NrGO NSs catalyst

**Fig. 1:**
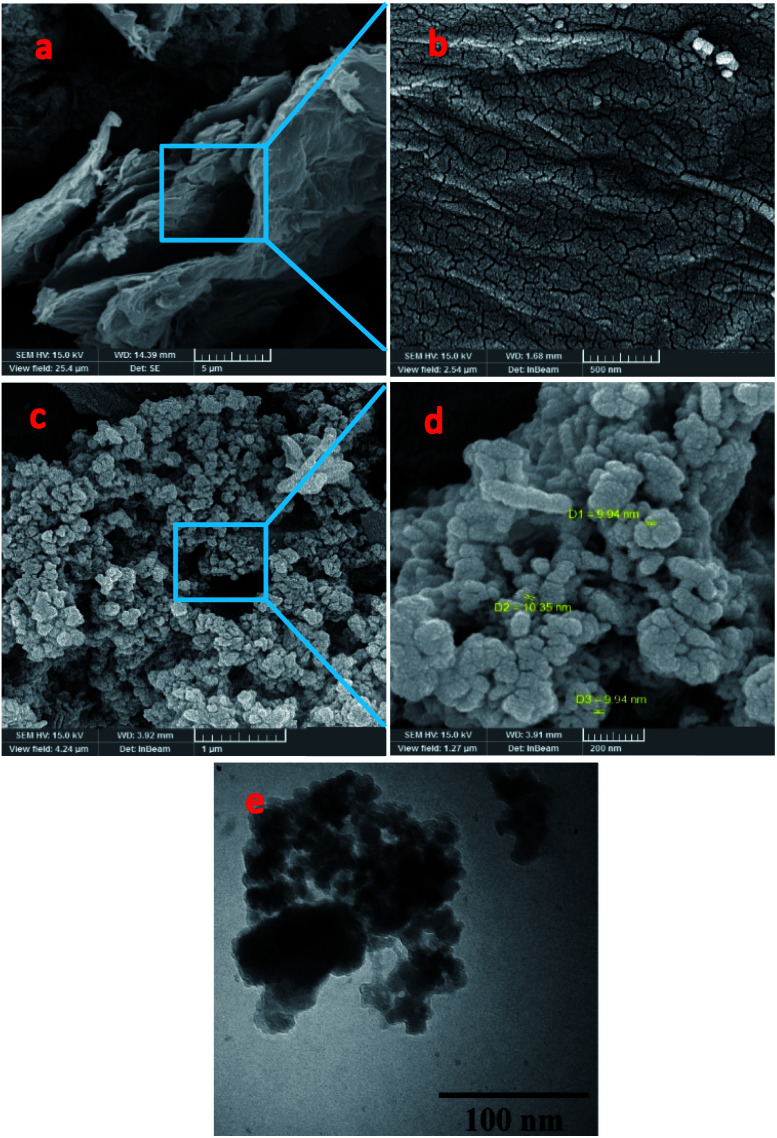
(a–d) FESEM images of (a and b) NrGO NSs and (c and d) PdCo NPs/NrGO NSs; (e) TEM image of PdCo NPs/NrGO NSs.

The morphology and structure of the synthesized samples (NrGO NSs and PdCo NPs/NrGO NSs) were identified by SEM, EDX, TEM, and XRD techniques. As evidenced from Fig. 1a and b, the flat and stacked multilayer sheets with wrinkled and folded features are present on the NrGO NSs surface. The strongly interconnection of these planner sheets are corroborated the retention of rGO morphology after nitrogen doping.^51^ The wrinkled graphene sheets are covered entirely with large quantities of PdCo NPs with an average diameter of around 10 nm, as presented in Fig. 1c and d. On the other hand, the compact exfoliated multilayers of NrGO NSs structures in PdCo NPs/NrGO NSs and also the uniform dispersion of metal NPs on the catalyst support affirm the solitary formation of PdCo NPs on the rough and planar NrGO NSs nanosheets. From the TEM image of PdCo NPs/NrGO NSs (Fig. 1e), it is clear that the metal nanoparticles are uniformly scattered on the NrGO NSs sheet with a narrow size range. This uniform dispersion may be ascribed as shifting the d-band centre's of supported Pd atoms to the Fermi level in the presence of nitrogen atoms.

The results from EDX analysis (as seen in Fig. S2†), are corroborated that the total metals content is 20 wt% and the weight ratio of Pd to Co is 1 : 1. Moreover, the elemental mapping images presented in Fig. S3† display that the bimetallic metal NPs are deposited homogeneously on the active sites of NrGO NSs.

**Fig. 2:**
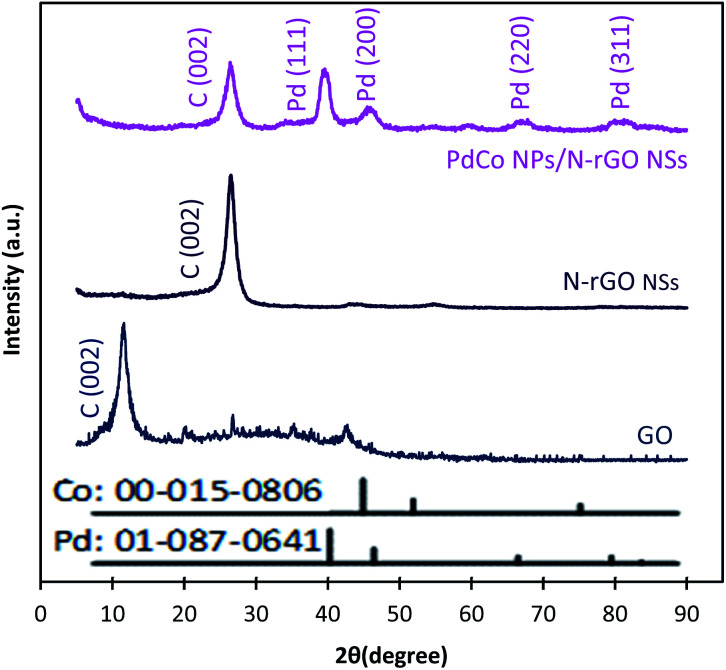
XRD patterns of GO, NrGO NSs, and PdCo NPs/NrGO NSs.

Typical XRD diffractograms of GO, NrGO NSs, and PdCo NPs/NrGO NSs samples are depicted in Fig. 2. In XRD pattern of GO, a strong sharp diffraction peak takes place at 2*θ* = 11.49° which is related to the basal plane (002) of graphitic structure (002);^50^ whereas, it is shifted to more positive values, *i.e.* 2*θ* = 26.45° in XRD patterns of NrGO NSs and PdCo NPs/NrGO NSs samples. According to the lattice parameters (*a*_fcc_) reports in the literature^52,53^ for rGO (0.37 nm) and NrGO NSs (0.34 nm), it is concluded that the crystallite size of rGO is decreased by nitrogen doping into GO framework. This means that the graphitic structure is more compacted after doping of nitrogen atoms.^54,55^ XRD diffractogram of PdCo NPs/NrGO NSs affirms the fcc crystalline structure of Pd with peaks at 40.15°, 46.80°, 68.29°, and 82.28° which are correspond to the (111), (200), (220), and (311) planes of Pd NPs (JCPDS No. 01-087-0641), respectively (Fig. 2).^56,57^ It is clear that the diffraction peaks of PdCo NPs/NrGP NSs are located between those of pure Pd and Co (JCPDS No. 00-015-0806) NPs and in the most cases shifted to large angles.

### Evaluation of electrocatalytic activity for PdCo NPs/NrGO NSs in an alkaline solution

**Fig. 3:**
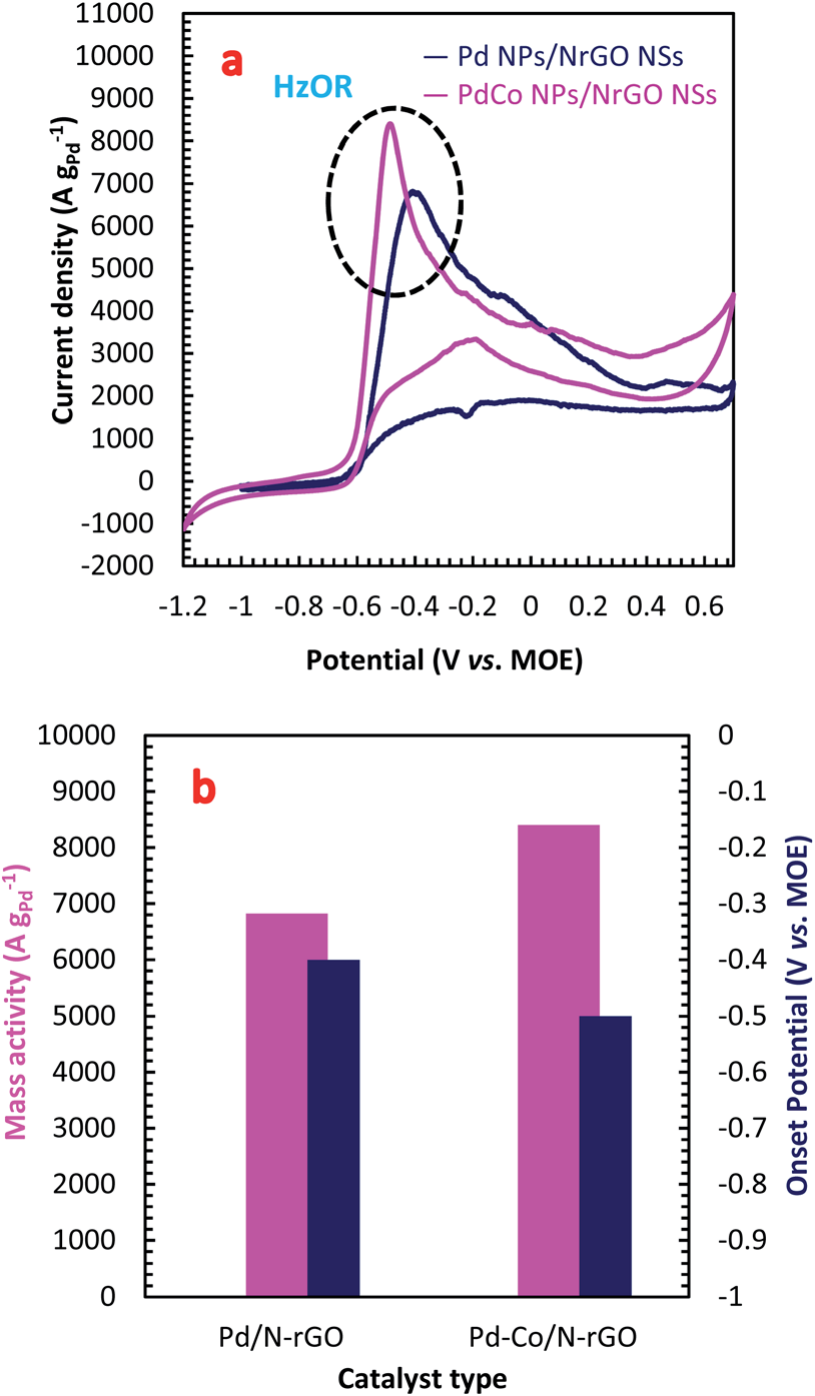
(a) The CV curves in an aqueous solution of 1 mol L^−1^ NaOH + 0.02 mol L^−1^ N_2_H_4_ under a constant sweep rate of 100 mV s^−1^; (b) the mass activity and onset potential for PdCo NPs/NrGO NSs and Pd/NrGO NSs under experiment conditions.

The electrocatalytic activity of the PdCo NPs/NrGO NSs and Pd/NrGO NSs samples towards HzOR were evaluated by CVs analysis in a binary aqueous (1 mol L^−1^ NaOH + 0.02 mol L^−1^ N_2_H_4_) solution at a sweep rate of 100 mV s^−1^ at room temperature under sweeping the potential from −1.2 to 0.7 V *vs.* MOE. The results are plotted in Fig. 3a. As presented in this figure, the hydrazine oxidation peaks for PdCo NPs/NrGO NSs and Pd/NrGO NSs appeared at a potential window of −0.6 to −0.3. Eqn (4) describes the HzOR taken place on the surface of synthesized catalysts.^58^

4 N_2_H_4_ + 4OH^−^ → N_2_ + 4H_2_O + 4e^−^

The absence of a faradaic process and also inactivity of NrGO NSs against HzOR are more proven by the finding of no obvious peak in CVs of NrGO NSs in the presence and absence of 0.02 mol L^−1^ N_2_H_4_ at a constant sweep rate of 100 mV s^−1^, as seen in Fig. S4a.†

From Fig. 3a, it can be observed that the PdCo NPs/NrGO NSs catalyst not only represents high current density (8399.76 A g^−1^) in comparison with the Pd/NrGO NSs (6821.23 A g^−1^) but also delivers a more negative value of the onset potential than other catalysts (Fig. 3b). This means that the bimetallic PdCo NPs have a more significant influence on the electrochemical reaction and improves the use of Pd for adsorption and oxidation of hydrazine compared to the monometallic Pd catalyst. This result suggests that the PdCo NPs/NrGO NSs electrocatalyst may be a favourable alternative anodic catalyst for HzOR in an alkaline water medium. The mass activities (MAs) of synthesized catalysts are also presented in Fig. 3b. It is clear that the MA value of the PdCo/NrGO is higher than that of Pd/NrGO in good consistence of ECSA values calculated for each catalyst.

To obtain more information about the high catalytic activity of PdCo NPs/NrGO NSs in comparison of Pd/NrGO NSs, their CVs were recorded a constant sweep rate of 100 mV s^−1^ in 1 mol L^−1^ NaOH and the results are plotted in Fig. S4b.† According to this figure, the hydrogen desorption and reduction of PdO formed on two electrocatalysts surface are appeared at the potential regions of −0.75 to −0.3 V in the forward sweeps and −0.2 to −0.4 V in the backward sweeps, respectively. The areas of PdO reduction in CVs presented in Fig. S4b† are used to compute the electrochemical surface area (ECSA) of each electrode. The ECSA is a good criterion for obtaining a full insight in regard with the catalyst electrochemical activity; so that, a high value of ECSA presents a high catalytic activity. More details about the ECSA calculation are also reported in the ESI.† The calculated ECSA values for the PdCo NPs/NrGO NSs and Pd/NrGO catalysts are 135.96 m^2^ g^−1^ and 63.67 m^2^ g^−1^, respectively. According to these values, it is expected that the PdCo NPs/NrGO NSs present a superior electrocatalytic performance against HzOR than that of Pd/NrGO NSs, as shown in practice.

**Fig. 4:**
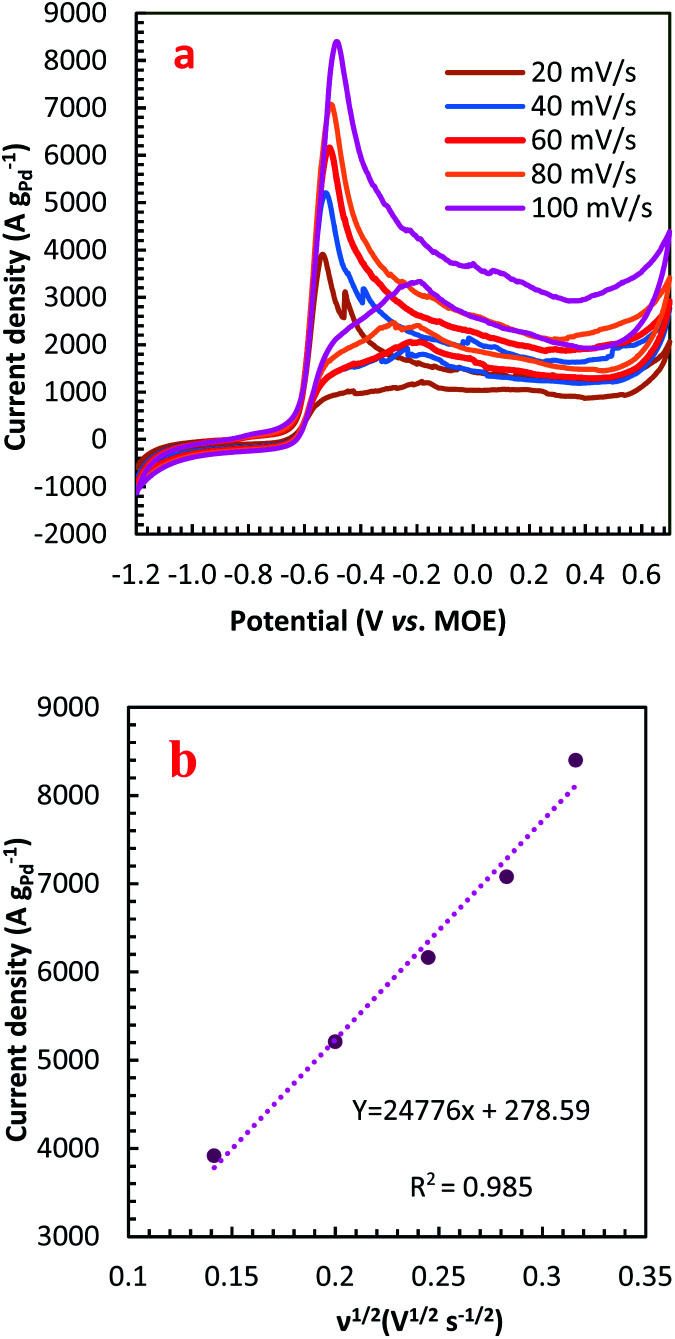
(a) The effect of sweep rate on the CVs of PdCo NPs/NrGO NSs in 1 mol L^−1^ NaOH + 0.02 mol L^−1^ N_2_H_4_; (b) the plot of HzOR peak current *vs. ν*^1/2^ for PdCo NPs/NrGO NSs.

For further illustration of the electrocatalytic properties of the PdCo NPs/NrGO NSs catalysts, CV tests were conducted at different sweep rates in the mixture of NaOH 1.0 M and N_2_H_4_ 0.02 M at ambient temperature, and the results are shown in Fig. 4a. It is clear that the *i*_p_ values are enhanced with an enhance the scan rate. According to eqn (5), the *i*_p_ value is commensurate to the square root of sweep rate (
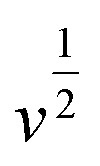
).^59^ From plotting the calculated *i*_p_ values *versus*
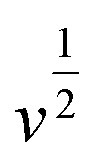
, a good linear relationship is obtained (as seen in Fig. 4b) which suggests the HzOR on the synthesized catalyst is a diffusion-controlled process.^60,61^5
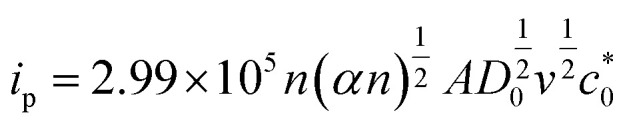
6
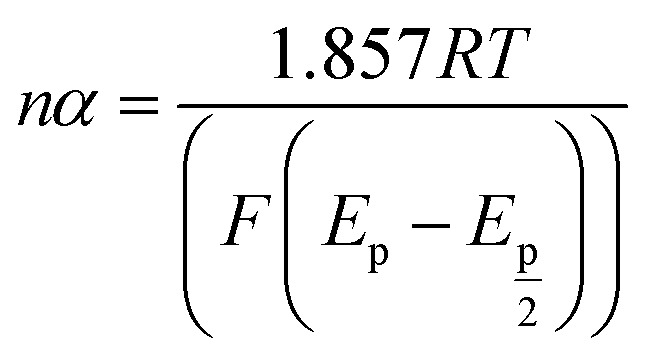


In eqn (5) and (6), *A*, *D*_0_, *E*_p,_ and *E*_p/2_ are the electrode geometrical surface area (cm^2^), diffusion coefficient, peak, and half-peak potential (V), respectively. 
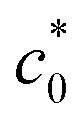
 refers to the bulk concentration of electroactive species^62^ which was 0.02 mol dm^−30^ in the present study.

Simultaneously, the peak potential tends toward a more positive voltage by increasing sweep rates, as described by eqn (7).^63^ This behavior is revealed that the hydrazine oxidation on the as-prepared catalyst is an irreversible electrochemical reaction.^64^7
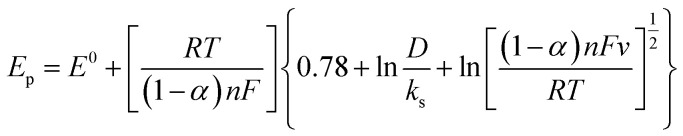
in this relation *R*, *T*, *α*, *F*, and *k*_s_, denote to the gas constant (8.314 J K^−1^ mol^−1^), temperature (K), charge transfer coefficient, Faraday constant (96 485 C mol^−1^), and the standard heterogeneous rate constant (cm s^−1^), respectively. *E*^0^ and *E*_p_ are standard and peak potential (V), respectively.

**Fig. 5:**
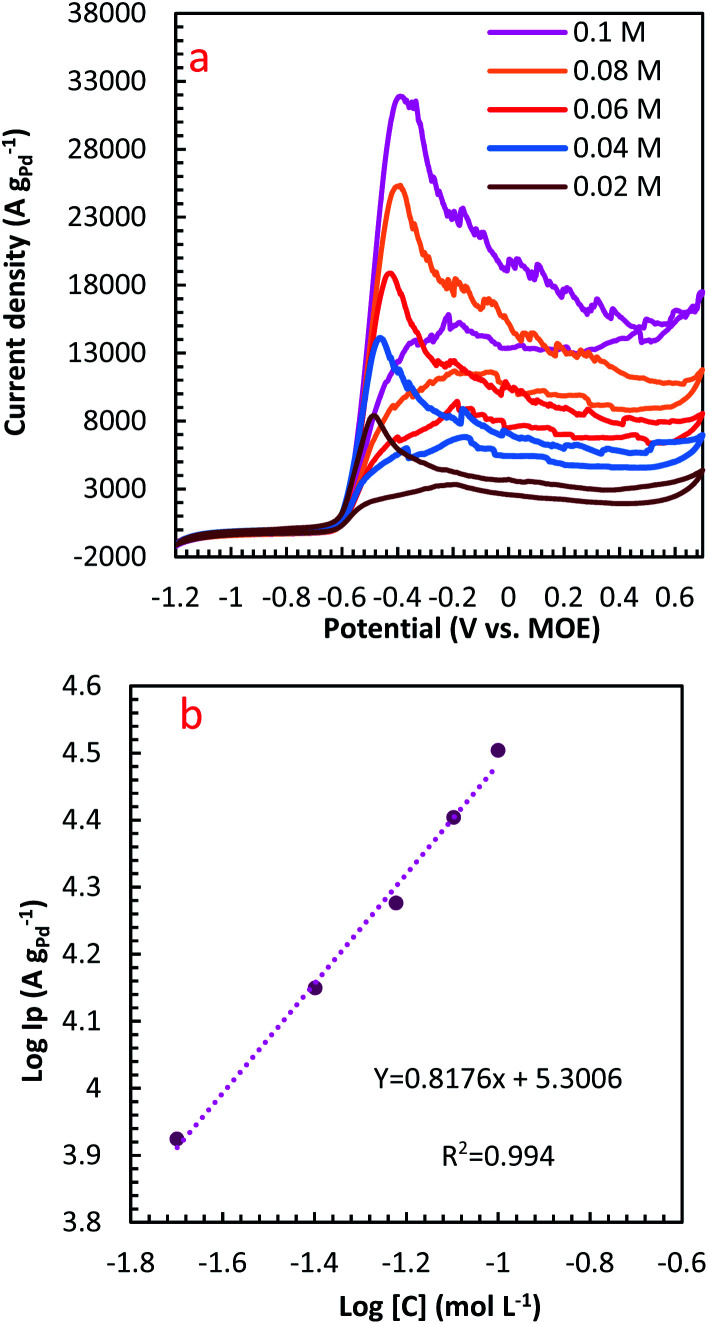
(a) The effect of N_2_H_4_ concentration on the CVs of PdCo NPs/NrGO NSs in 1 mol L^−1^ NaOH at a constant sweep rate of 100 mV s^−1^; (b) the plot of log *C*_N_2_H_4__–log *i*_p_ for PdCo NPs/NrGO NSs.

The influence of N_2_H_4_ concentration, [N_2_H_4_], was studied on cyclic voltammograms of PdCo NPs/NrGO NSs in an aqueous solution of 1 mol L^−1^ NaOH. It is obvious that the *i*_p_ value is increased with increasing [N_2_H_4_] (Fig. 5). Besides, the potential of N_2_H_4_ oxidation tends towards a more positive value by enhancing [N_2_H_4_] indicating a diffusion-controlled process. By plotting log *i*_p_ values in terms of log[N_2_H_4_], a straight linear relation is achieved (Fig. 5b), in which the slope of this linear plot corresponds to the reaction order of N_2_H_4_ oxidation on the PdCo NPs/NrGO NSs. The relation between *i*_p_ and [N_2_H_4_] values are summarized as follows:

8 Rate = *i*_p_ = *k*[N_2_H_4_]^*β*^

9 log *i*_p_ =  log *k* + *β*log[N_2_H_4_]

where *k*, [N_2_H_4_] and *β* relate to the constant equation, the bulk [N_2_H_4_], and the order of reaction, respectively. The calculated reaction order value for the PdCo NPs/NrGO NSs was 0.82. This value is close to a regular value of 1 which reveals a first-order reaction for HzOR in good agreement with the ones reported in literature.^65^

**Fig. 6:**
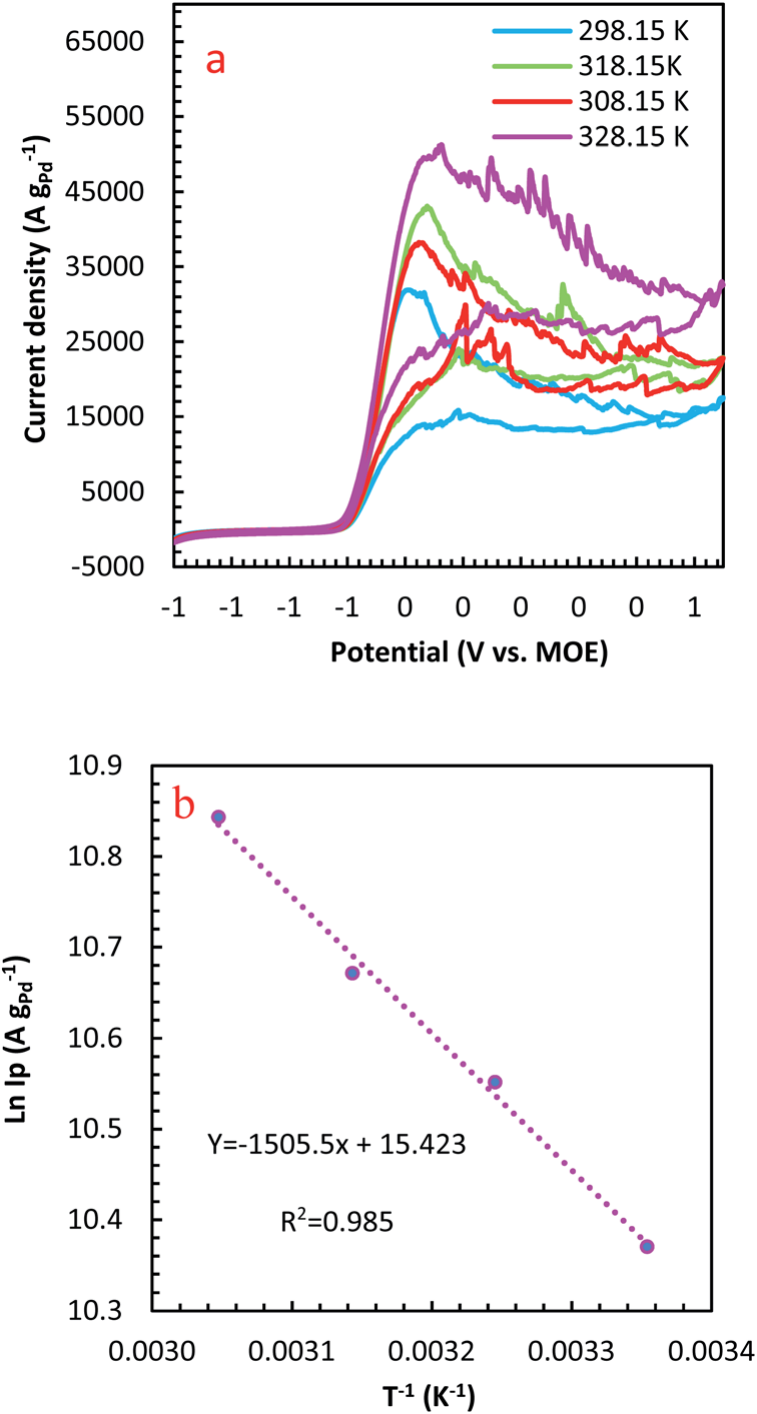
(a) The influence of temperature on the CV curves of PdCo NPs/NrGO NSs in 1 mol L^−1^ NaOH + 0.1 mol L^−1^ N_2_H_4_ at a constant sweep rate of 100 mV s^−1^; (b) the Plot of ln *i*_p_*vs.* 1/*T* for PdCo NPs/NrGO NSs.

The temperature usually presents a remarkable influence on the charge and mass transfer. So, it is essential to survey the temperature impact on the electrochemical behaviour of PdCo NPs/NrGO NSs catalyst in an alkaline solution containing 1 mol L^−1^ N_2_H_4_. Fig. 6a shows CVs recorded at 100 mV s^−1^ under four typical temperatures, *i.e.* 25, 35, 45, and 55 °C. From Fig. 6b, it can be concluded that the CV currents are increased by increasing temperature, which suggests a high electrochemical activity at high temperatures. This means that the charge transfer in electrode/electrolyte interface and also mass transfer into electrolyte solution are increased by increasing temperature. The variation of the CVs allows us to determine activation energy (*E*_a_) in a broad interval of potentials where characteristic oxidation peaks emerged. The *E*_a_ value is computed with the Arrhenius equation (eqn (10))^66^ and the value was 12.51 kJ mol^−1^.10
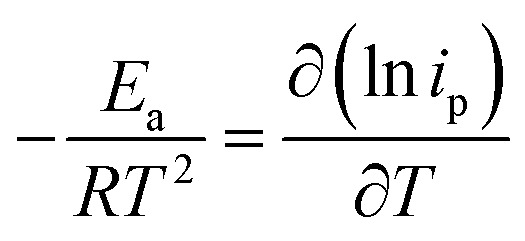


A lower value of *E*_a_ in comparison with those reported in literature^67,68^ illustrated that the HzOR on the PdCo NPs/NrGO NSs is occurred easily because of its large specific surface area and massive catalytic active sites.

**Fig. 7:**
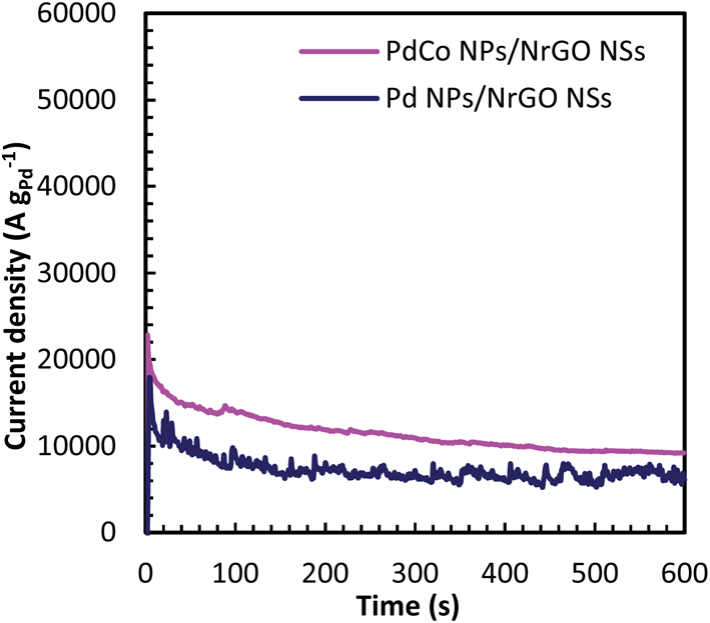
Chronoamperometry curves of PdCo NPs/NrGO NSs and Pd/NrGO in 1 mol L^−1^ NaOH + 0.1 mol L^−1^ N_2_H_4_ at −0.5 V.

Chronoamperometry (CA) is known as a time-dependent method that measures the current density as a function of time. It can be utilized for determining the current–time dependency of diffusion-controlled processes that taken place on the electrode surface. Fig. 7 presents current–time curves of PdCo NPs/NrGO NSs and Pd/NrGO NSs electrocatalysts in mixture of 1 mol L^−1^ NaOH and 0.1 mol L^−1^ N_2_H_4_ at −0.5 V *vs.* MOE. The higher current density obtained after 600 s for PdCo NPs/NrGO NSs (9200 A g^−1^) than that of Pd/NrGO NSs (6800 A g^−1^) is more confirm its good performance.

**Fig. 8:**
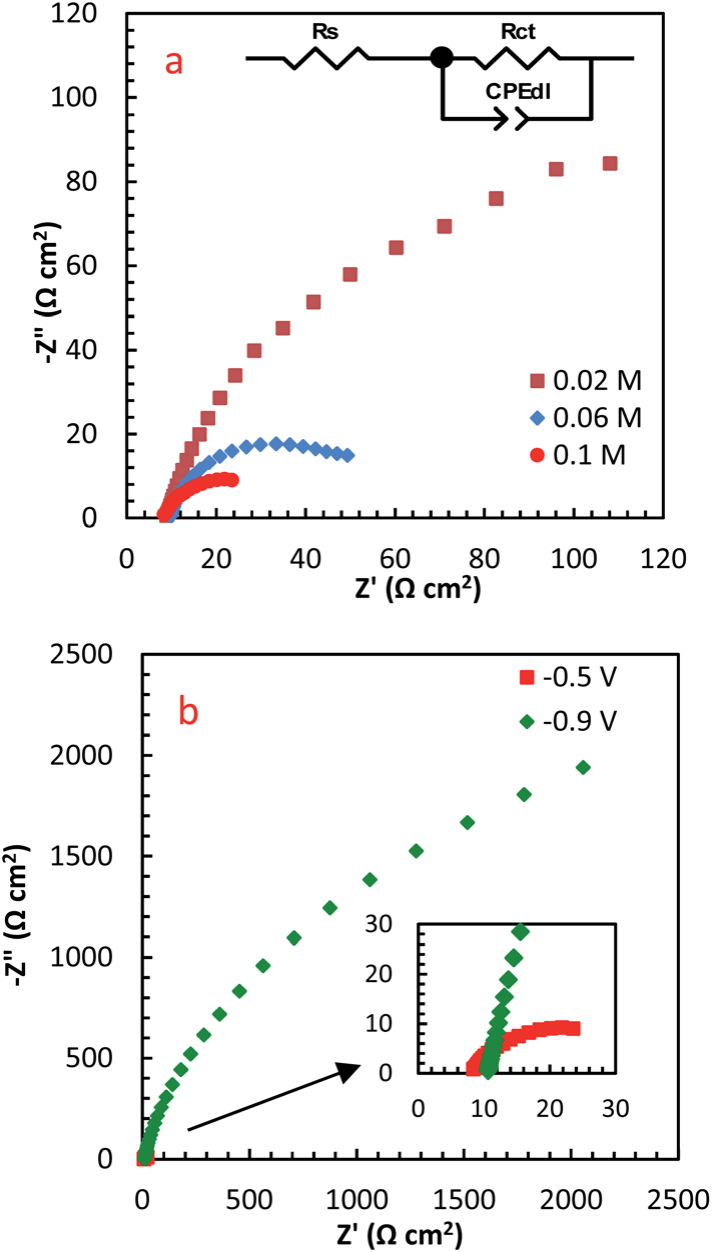
The influence of (a) N_2_H_4_ concentration, and (b) potential on EIS spectra of PdCo NPs/NrGO NSs.

EIS is a high-sensitive technique for probing the specifications of surface reconstructed electrodes. Fig. 8a represents the EIS results of PdCo NPs/NrGO NSs electrode in mixture of 1 mol L^−1^ NaOH and *y* mol L^−1^ N_2_H_4_ (*y*: 0.02, 0.06 and 0.1) at two potentials (−0.5 and −0.9 V). The electrical equivalent circuit employed to fit impedance information is displayed in the inset of Fig. 8a. In this figure, *R*_ct_, *R*_s,_ and CPE_d_ correspond to the charge transfer resistance, electrolyte resistance, and the constant phase element in the less-than-ideal behaviour of the electrical double layer, respectively. One semicircle in the EIS spectra relates to the electrochemical reactions taken place on the catalyst surface. The intersection point between the horizontal axes with this semicircle at high frequency and low-frequency regions is utilized for estimation of the *R*_s_ and *R*_ct_ values, respectively.

Table 1 summarizes the EIS results of PdCo NPs/NrGO NSs electrode under different operational conditions. The results indicated that the capacitive arc diameter, as a charge transfer resistance factor, is decreased very significantly with increasing [N_2_H_4_]. That is, the HzOR occurred on the catalyst surface is accelerated significantly by an enhance the hydrazine concentration (as presented in Fig. 8a). A decrease in the diameter of the semicircle is also seen when potential increases from −0.9 to −0.5 V (Fig. 8b), *i.e.* N_2_H_4_ is fast-oxidized at −0.5 V. This result is in good consistent with the CVs.

**Table 1:** The values of charge transfer resistances for hydrazine oxidation on the PdCo NPs/NrGO NSs catalyst at various concentrations of hydrazine and voltages

[N_2_H_4_] (mol L^−1^)	Potential (V *vs.* MOE)	Temperature (°C)	*R*_ct_ (Ω cm^2^)
0.02	−0.5	25	213.02
0.06	−0.5	25	77.69
0.10	−0.5	25	36.13
0.10	−0.9	25	3729

### Fuel cell performances

**Fig. 9:**
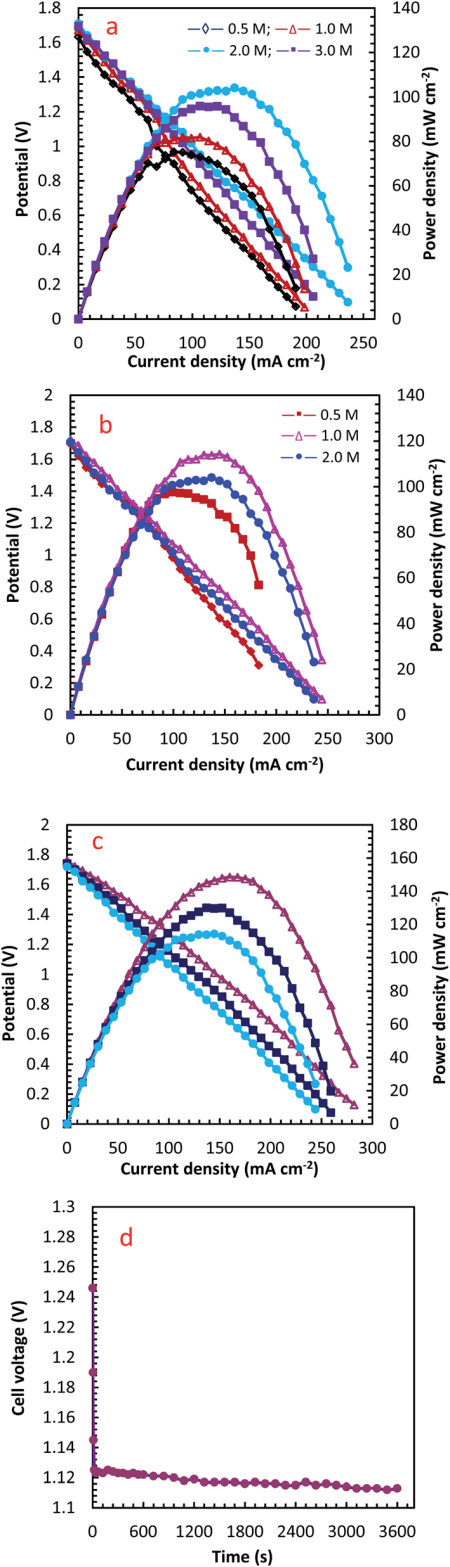
The effect of operation condition on the performance of DHzHPFC designed with PdCo NPs/NrGO NSs (1.0 mg cm^−2^) as an anode and Pt/C (0.5 mg cm^−2^) as a cathode: (a) H_2_O_2_ concentration, (b) N_2_H_4_ concentration, and (c) temperature; (d) stability test for PdCo NPs/NrGO NSs at a discharging current of 90 mA cm^−2^.

A single cell with the PdCo NPs/NrGO NSs (1.0 mg cm^−2^) as an anode and Pt/C (0.5 mg cm^−2^) as a cathode was designed and used to investigate cell performance by recording the polarization and the power density curves. In these tests, the mixtures of (0.5 mol L^−1^ H_2_SO_4_ + *i* mol L^−1^ H_2_O_2_ (*i*: 0.5, 1.0, 2.0 and 3.0)) and (2.0 mol L^−1^ NaOH + *j* mol L^−1^ N_2_H_4_ (*j*: 0.5, 1.0, and 2.0)) were used as an oxidant and fuel at three temperatures (25, 45 and 60 °C). The optimum conditions in which the cell performance is maximum were determined and reported in Table 2. The obtained results are plotted in Fig. 9a–c.

**Table 2:** The comparison of direct hydrazine–hydrogen peroxide fuel cell performance under different experimental cell conditions

Anode	Cathode	Membrane	Anolyte	Catholyte	T (°C)	MPD (mW cm^−2^)	Ref.
Pt_53_Cu_47_/C (0.5 mg cm^−2^)	Pt/C (20 wt%) (1.0 mg cm^−2^)	Tokuyama	KOH 1.0 M + N_2_H_4_ 1.0 M	O_2_ flow rate: 30 SCCM	80	56.1	75
Ni_0.6_Co_0.4_ nanosheets (1.4 mg cm^−2^)	Pt/C (40 wt%)	Nafion 115	KOH 4.0 M + N_2_H_4_ 20.0 wt%	H_2_O_2_ 20.0% + H_2_SO_4_ 0.5 M	80	107.1	76
Pd/CNT (1.0 mg cm^−2^)	Pt/C (0.25 mg cm^−2^)	Nafion 117	NaOH 1.0 M + N_2_H_4_ 2.0 M	O_2_ flow rate: 150.0 mL min^−1^	60	110	77
Co@Au/C (1.0 mg cm^−2^)	Au/C (1.0 mg cm^−2^)	Nafion 117	NaOH 2.0 M + N_2_H_4_ 2.0 M	H_2_O_2_ 2.0 M + H_2_SO_4_ 0.5 M	60	122.8	9
MoC_*x*_–NC (1.0 mg cm^−2^)	Pt/C (1.0 mg cm^−2^)	KOH-doped PBI	KOH 6.0 M + N_2_H_4_ 0.5 M	O_2_ flux: 0.2 slpm	80	158.26	78
Ni@Pd/rGO (1.0 mg cm^−2^)	Pt/C (0.5 mg cm^−2^)	Nafion 117	NaOH 2.0 M + N_2_H_4_ 1.0 M	H_2_O_2_ 2.0 M + H_2_SO_4_ 0.5 M	60	204.8	23
PdCO NPs/NrGO NSs (1.0 mg cm^−2^)	Pt/C (0.5 mg cm^−2^)	Nafion 117	N_2_H_4_ 1.0 M + NaOH 2.0 M	H_2_O_2_ 2.0 M + H_2_SO_4_ 0.5 M	45 60	130.10 148.58	This work

The influence of different H_2_O_2_ concentrations ([H_2_O_2_]) on the cell performance is presented in Fig. 9a. According to the occurred reaction on the electrode surface and the Nernst equation (eqn (11)), the open circuit voltage (OCV) is enhanced by enhancing [H_2_O_2_]. Also, the cell performance is increased from 75.31 to 81.88 and 104.04 mW cm^−2^ by increasing the [H_2_O_2_] from 0.5 to 1.0 and 2.0 mol L^−1^, respectively. By more increasing [H_2_O_2_] from 2.0 mol L^−1^ to 3.0 mol L^−1^, however, the maximum power density (MPD) is reduced to 95.85 mW cm^−2^. The possible reason for this observation may be related to the rapid chemical decomposition of H_2_O_2_ at high concentrations, attachment of gas bubbles from decomposition, and crossover of H_2_O_2_ from the membrane into a thicker solution.^23^ So, the value of 2.0 mol L^−1^ is selected as an optimum [H_2_O_2_].11
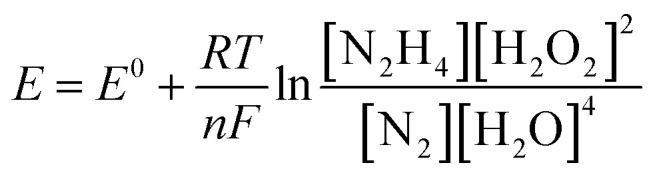


The influence of [N_2_H_4_] on cell efficiency was also assessed and the results are presented in Fig. 9b. As presented in this figure, the OCV values of DHzHPFC is changed by enhancing [N_2_H_4_].^69,70^ When [N_2_H_4_] is enhanced from 0.5 to 1.0 mol L^−1^, the anode potential and cell OCV are increased. That is, the fuel diffusion and the oxidation kinetics of N_2_H_4_ are improved by increasing [N_2_H_4_], as expected from eqn (11). Although the fuel usage efficiency is enhanced by enhancing the [N_2_H_4_], its crossover is intense. Therefore, it is expected that the cell performance is initially increased and then reduced by further enhancing [N_2_H_4_].^9,71,72^ As expected, the power density of DHzHPFC is raised from 97.62 to 114.31 mW cm^−2^ with an increase the [N_2_H_4_] from 0.5 to 1.0 mol L^−1^ and followed then by decreasing at 2.0 M to 104.04 mW cm^−2^. Decreasing the MPD values with an increase [N_2_H_4_] from 1.0 to 2.0 mol L^−1^ can be related to increasing of hydrazine crossover and its hydrolysis. So, the value of 1.0 mol L^−1^ is selected as an optimum [N_2_H_4_].

Fig. 9c presents typical polarization and power density curves of DHzHPFC at different cell temperatures. It is clearly obvious that the MPDs are enhanced from 114.31 to 130.10 and 148.58 mW cm^−2^ by increasing temperature from 25 to 45 and 60 °C, respectively. This behaviour can be related to increase kinetics of anodic and cathodic reactions and improving electrolyte conductivity at high temperature.^73,74^Table 2 collects the MPD value of the synthesized catalyst and those reported in literature. As seen in this table, the MPD value of the PdCo NPs/NrGO NSs is comparable with the other bimetallic alloying anodic catalysts, *i.e.* the synthesized catalyst can be considered as an interesting and low-priced candidate for DHzHPFC.

To determine of electrochemical stability of the PdCo NPs/NrGO NSs nanocatalysts, the cell OCV were investigated as a function of time using an anolyte (2.0 mol L^−1^ NaOH + 0.1 mol L^−1^ N_2_H_4_) and catholyte (0.5 mol L^−1^ H_2_SO_4_ + 2.0 mol L^−1^H_2_O_2_) at 90 mA cm^−2^ and 25 °C. The result is presented in Fig. 9d. Oxygen and hydrogen are produced as from H_2_O_2_ decomposition in the cathodic compartment and N_2_H_4_ hydrolysis in the anodic compartment, respectively. These products lead to fluctuations in the cell potential, as seen in Fig. 9d. The produced gas bubbles can also amass on the electrode surface and block the transference of N_2_H_4_ or H_2_O_2_ solutions and leading to an instant loss of performance. Following a decay in potential during the first few seconds, however, the cell potential can be rapidly restored to its normal discharge potential, as displayed in Fig. 9d. Although the long-term durability of the prepared catalyst and DHzHPFCs needs some further investigation, the results obtained in this work indicated that the catalytic activity of PdCo NPs/NrGO NSs against N_2_H_4_ oxidation is relatively stable in DHzHPFCs.

## Conclusions

In the present work, PdCo NPs/NrGO NSs were prepared as high-performance and low-cost anodic nanocatalyst by using the electroless method and subsequently characterized using FT-IR, XRD, SEM, EDX, and TEM. The FT-IR and XRD spectroscopic studies confirm the reduction of oxygen functionalities in GO and also doping of nitrogen atoms into GO framework. The morphological images indicated that the PdCo NPs were more uniformly scattered on the NrGO NSs with a narrow crystalline size of about 10 nm. The electrochemical behavior of the synthesized electrocatalyst was studied by half-cell tests and the hydrazine oxidation current density of 8399.76 A g^−1^ was obtained for PdCo NPs/NrGO NSs. This means that the PdCo NPs/NrGO NSs catalyst could be acted as a superior catalyst toward HzOR. Moreover, a low value of activation energy of PdCo NPs/NrGO NSs (12.51 kJ mol^−1^) showed that this catalyst was more suitable than that of Pd/NrGO NSs. Finally, the cell performance of a prepared anodic catalyst was investigated in a single fuel cell under various temperatures, and also H_2_O_2_ and N_2_H_4_ concentrations. Under optimum conditions (*i.e.*, 0.1 mol L^−1^ N_2_H_4_, 2 mol L^−1^ H_2_O_2,_ and 60 °C), the MPD value of PdCo NPs/NrGO NSs was achieved at about 148.58 mW cm^−2^. This result and the high durability of PdCo NPs/NrGO NSs in comparison with Pd/NrGO NSs suggested that the PdCo NPs/NrGO NSs can be economical, powerful, and reliable replacement for precious noble metal-based anode catalysts for direct hydrazine–hydrogen peroxide fuel cells.

## Author contributions

Mir Ghasem Hosseini: supervision, conceptualization, validation. Vahid Daneshvari-Esfahlan: conceptualization, investigation, writing–original draft preparation, formal analysis. Sigrid Wolf: reviewing and editing. Viktor Hacker: supervision, reviewing and editing.

## Conflicts of interest

There are no conflicts of interest to declare.

## Supplementary Material

RA-011-D1RA07099A-s001
