# Supplementary material for: Cobalt-modified palladium nanocatalyst on nitrogen-doped reduced graphene oxide for direct hydrazine fuel cell
Source: RSC Adv. 2021 Dec 8;11(62):39223–32. doi: 10.1039/d1ra07099a (PMC9044457; doi:10.1039/d1ra07099a)
Supplement: RA-011-D1RA07099A-s001 [file RA-011-D1RA07099A-s001.pdf]

Electronic Supplementary Material (ESI) for RSC Advances.
This journal is © The Royal Society of Chemistry 2021

Supporting information

Cobalt-modified palladium nanocatalyst on nitrogen-doped reduced graphene oxide for direct hydrazine fuel cell

Mir Ghasem Hosseini[*,a], Vahid Daneshvari-Esfahlan [a, b], Sigrid Wolf [b], Viktor Hacker [*,b]

[a] Electrochemistry Research Laboratory, Department of Physical Chemistry, Faculty of Chemistry, University of Tabriz, Tabriz 51666-16471, Iran

[b] Institute of Chemical Engineering and Environmental Technology, Graz University of Technology, Inffeldgasse 25/C, 8010 Graz, Austria

Synthesis of graphene oxide (GO). GO was fabricated by the modified Hummers method, as previously described. [1, 2] A natural graphite powder (5.0 g) was added to a flask containing 120 mL of sulfuric acid solution in an ice bath with a subsequent addition of $NaNO_3$ (2.5 g) and $KMnO_4$ (15.0 g) to the suspension without stirring. After the ice bath was ejected, the solution was stirred at 50 ℃ for 12 h. Then, 150 mL of Millipore water was added gradually to the mixture and refluxed at 98 ℃ for 24 h. following on from this aqueous solution of H_2O_2 (50.0 mL) was added to this mixture to convert the insoluble manganese species to Mn^{2+} ions. Then, the mixture was then centrifuged using HCl to obtain the GO. The GO was rinsed with Millipore water several times, and a viscous brown dispersion was formed, filtrated, and finally dried at 65 ℃ under vacuum.

Pre-treatment of Nafion 117. For this goal, a Nafion 117 membrane was immersed for 1 h in 3 wt % boiling H_2O_2 to remove any organic impurities and then washed with Millipore water. Following on from this it was again immersed in hot H_2SO_4 (1.0 mol L^{-1}) for other 1 h to remove any metallic impurities and also to convert the membrane fully to the H^+ form and then boiled in Millipore water for 1 h. This last treatment was repeated twice to completely remove any traces amount of H_2SO_4 in the membrane. [3] After this procedure the anode, cathode and pre-treated Nafion 117 membrane were sandwiched together and hot pressed at 65 bar and 130 ℃ for 3 min. To humidify and modify the Nafion 117 membrane into the Na^+ form, 2.0 mol L^{-1} NaOH solution was circulated for 3 h at 40 ℃ before every test in the test fuel cell.

FT-IR study. According to Fig. S1, the band observed at 1728 cm^{-1} corresponds to the C=O stretching of the existing carboxylic groups at the edges of the GO sheets. The C=C stretching vibrations of GO was assigned to the band found at 1624 cm^{-1}. Two peaks observed at 1220 cm^{-1} and 1085 cm^{-1} represent the C-O stretching vibrations of carboxyl and alkoxy groups, respectively. [4, 5] The intensive band appeared at 3400 cm^{-1} in each spectrum specifies the O-H stretching in the hydroxyl groups. The reduction of GO is ensured from the vanishing of C=O and C-O stretching vibrations in spectrum of NrGO NSs, which confirms the removal of oxygen functional groups. The presence of nitrogen atoms in the NrGO NSs catalyst support is certified from the bands appeared at 1187 cm^{-1} which attributes to C-N bonds (Fig. S1). Also, the superposition of C=C and C=N vibrations results in shifting in-plane vibration of C=C from 1624 to 1556 cm^{-1}. This downshifted peak may be related to a favorable π-π interactions between the GO and nitrogen atoms. These evidences confirm embedding of nitrogen-containing groups into rGO and successfully synthesize of NrGO NSs. [6, 7]

EDX analysis. The EDX data of PdCo NPs/NrGO NSs catalyst were collected to determine the weight percentage of each element present in catalyst and the results were as: C (44.00 wt %), N (13.66 wt %), O (22.14 wt %) Pd (11.27 wt %) and Co (8.93 wt %), as seen in Fig. S2. Based on the EDX result, the total metals content is 20 wt % and the weight ratio of Pd to Co is 1:1.

Calculation of ECSA values for PdCo NPs/NrGO NSs and Pd NPs/NrGO NSs catalysts. For the calculation of the ECSA values for two synthesized catalysts, it is necessary to record their CV curves at a constant sweep rate of 100 mV s^{-1} in 1.0 mol L^{-1} NaOH, as shown in Fig. S4b. As seen in this figure, for each CV profile, four major regions may be observed which are ascribed as the

* Corresponding author
Email: mg-hosseini@tabrizu.ac.ir, Tel. +98 41 3339 3138
Email: viktor.hacker@tugraz.at, Tel. +43 316 873 7461

hydrogen adsorption/desorption (I, IV), formation of PdO (II), and PdO reduction (III) regions. The adsorption/desorption peak current densities of hydrogen are at the potential area -0.75 to -0.3 V. Additionally, the desorption peak of hydrogen for the PdCo NPs/NrGO NSs catalyst is moved distinctly to lower potentials suggesting that the hydrogen adsorption strength on the Pd surface is weakened by its alloying with Co metal. [8] It can be observed that the oxidation of Pd surfaces has taken place in the anodic sweep at potentials above 0.35 V. In the backward sweep, the PdO reduction can be clearly observed with a peak position at -0.2 to -0.4 V. Obviously, the adsorption/desorption of hydrogen and formation and reduction of palladium (II) oxide peaks on the PdCo NPs/NrGO NSs catalyst are stronger than that of Pd/NrGO NSs.

The ECSA values of both synthesized catalysts can be derived from CVs (Fig. S4b) by integrating the charge on PdO reduction regions, according to eqn (S1).

$$ECSA = \frac{Q_{PdO}}{(0.405 \times [Pd])} \qquad \text{(S1)}$$

here Q_{PdO} relates to the required coulombic charge for the reduction of PdO (mC). The value of 0.405 as a proportionality constant is related to the required charge for reduction of monolayer PdO ($mC.cm^{-2}$). [Pd] is the Pd loading on the catalyst surface (mg). The ECSA values for PdCo NPs/NrGO NSs and Pd/NrGO NSs catalysts are 135.96 and 63.67 $m^2\ g^{-1}$, respectively. It can be recognized that the ECSA value on PdCo NPs/NrGO NSs is 2.13 times higher than that in Pd/NrGO NSs. This observation may be suggested that the PdCo NPs/NrGO NSs catalyst has a superior electrocatalytic performance than that of Pd/NrGO NSs. On the other hand, the number of electrochemically active sites per gram of the PdCo NPs/NrGO NSs is greater compared to Pd/NrGO NSs; so, it is thus expected that PdCo NPs/NrGO NSs will shows a higher catalytic performance against HzOR in an alkaline medium, because this increases its chance to contact the reactant.

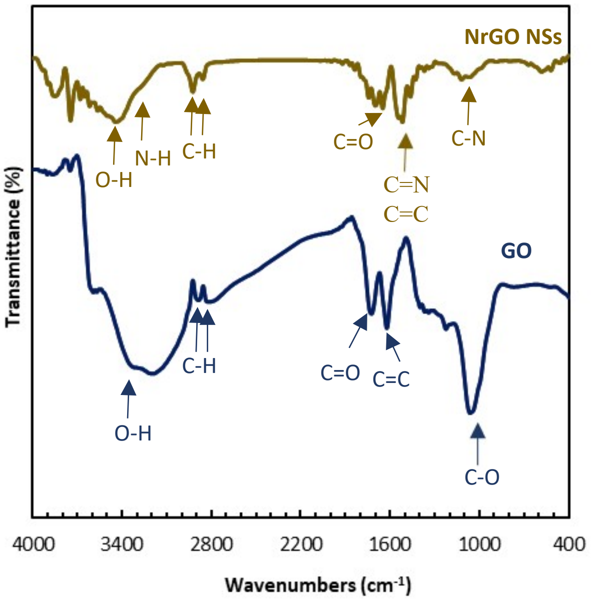

Fig. S1 FT-IR spectra of GO and NrGO NSs. [9]

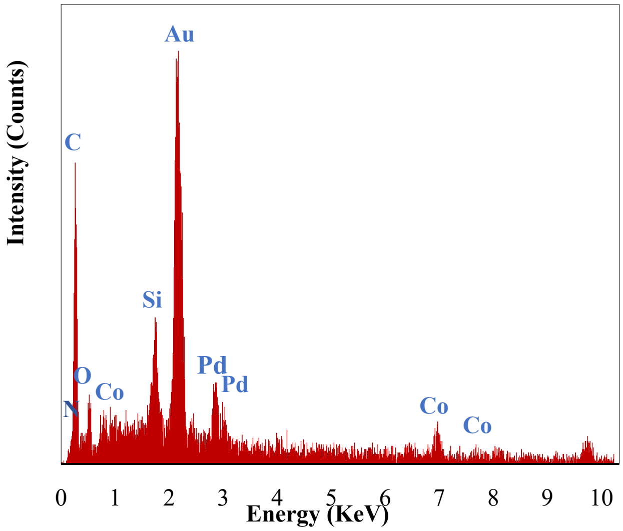

Fig. S2 EDX analysis of PdCo NPs/NrGO NSs. [9]

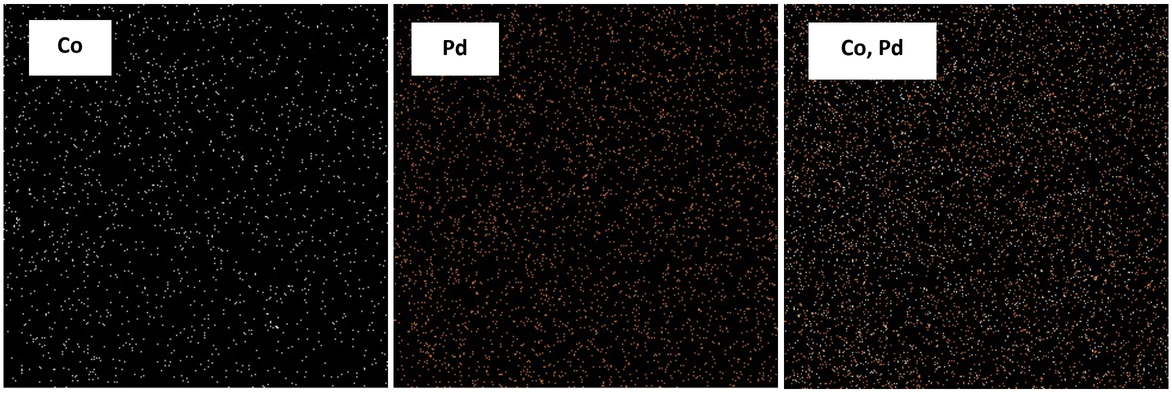

Fig. S3 The elemental mapping images of each element in PdCo NPs/NrGO NSs. [9]

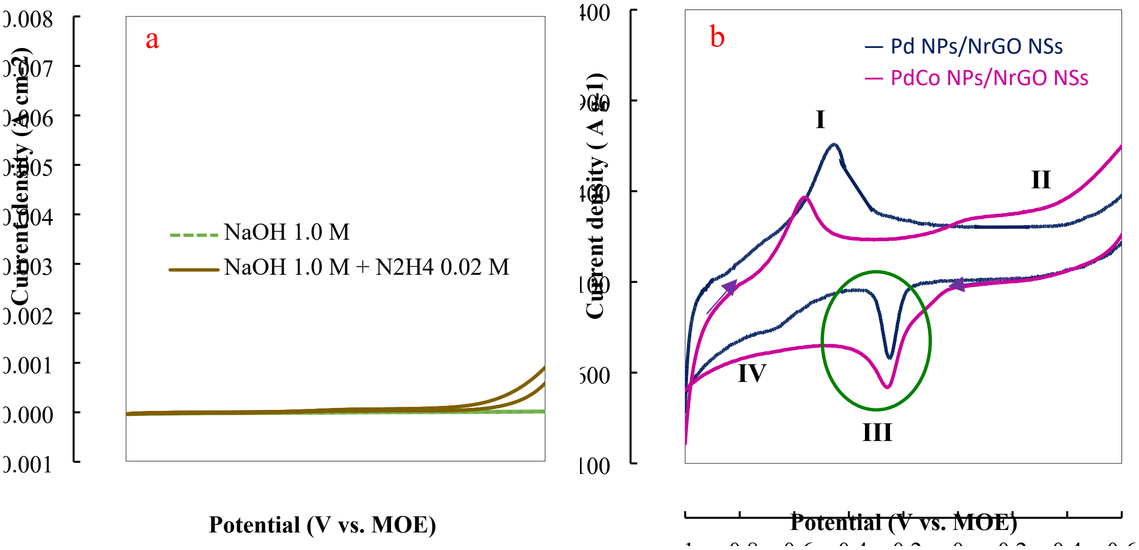

Fig. S4 (a) The CV curves in the NaOH 1.0 M under a constant scan rate of 100 mV s^{-1} (a) for NrGO NSs in the presence and absence of 0.02 mol L^{-1} N_2H_4, (b) for Pd NPs/NrGO NSs and PdCo NPs/NrGO NSs in the absence of N_2H_4.

References

1. M. Hirata, T. Gotou, S. Horiuchi, M. Fujiwara and M. Ohba, *Carbon*, 2004, **42**, 2929-2937.
2. W. S. Hummers and R. E. Offeman, *Journal of the American Chemical Society*, 1958, **80**, 1339-1339.
3. V. A. Paganin, E. A. Ticianelli and E. R. Gonzalez, *Journal of Applied Electrochemistry*, 1996, **26**, 297-304.
4. H.-L. Guo, X.-F. Wang, Q.-Y. Qian, F.-B. Wang and X.-H. Xia, *ACS Nano*, 2009, **3**, 2653-2659.
5. C. Zhu, S. Guo, Y. Fang and S. Dong, *ACS Nano*, 2010, **4**, 2429-2437.
6. Y. Xue, J. Liu, H. Chen, R. Wang, D. Li, J. Qu and L. Dai, *Angewandte Chemie International Edition*, 2012, **51**, 12124-12127.
7. X. Mu, B. Yuan, X. Feng, S. Qiu, L. Song and Y. Hu, *RSC Advances*, 2016, **6**, 105021-105029.
8. H. Xu, B. Yan, S. Li, J. Wang, C. Wang, J. Guo and Y. Du, *ACS Sustainable Chemistry & Engineering*, 2018, **6**, 609-617.
9. M. G. Hosseini, V. Daneshvari-Esfahlan, S. Wolf and V. Hacker, *ACS Applied Energy Materials*, 2021, DOI: 10.1021/acsaem.1c00876.
